# Theta-band EEG functional connectivity during emotional music in disorders of consciousness: wPLI differences between MCS and UWS

**DOI:** 10.3389/fneur.2026.1777525

**Published:** 2026-03-19

**Authors:** Sifan Wang, Chen Chen, Xintong Zhang, Shuang Zheng, Jinying Han, Yajuan Hu

**Affiliations:** 1 Department of Neurology, The First Affiliated Hospital of Anhui Medical University, Anhui Medical University, Hefei, China; 2 Anhui Province Key Laboratory of Cognition and Neuropsychiatric Disorders, The First Affiliated Hospital of Anhui Medical University, Hefei, China; 3 Collaborative Innovation Centre of Neuropsychiatric Disorder and Mental Health, Hefei, China; 4 School of Mental Health and Psychological Sciences, Anhui Medical University, Hefei, China; 5 Institute of Artificial Intelligence, Hefei Comprehensive National Science Center, Hefei, China; 6 Anhui Institute of Translational Medicine, Hefei, China

**Keywords:** disorder of consciousness, electroencephalography, emotional music stimulation, theta-band, weighted phase lag index

## Abstract

**Background:**

With the increasing number of patients with disorders of consciousness (DOC), accurate diagnosis—particularly differentiation between the minimally conscious state (MCS) patients and the unresponsive wakefulness syndrome (UWS) patients—is critical for subsequent treatment planning.

**Objective:**

To investigate differences in theta-band electroencephalography (EEG) functional connectivity between MCS and UWS patients during emotional music stimulation, and to explore their responsiveness to different emotional music conditions.

**Methods:**

Forty-eight DOC patients were enrolled (MCS, *n* = 32; UWS, *n* = 16). EEG was recorded during the resting state and while listening to four emotion-specific music conditions (sad, fearful, happy, and peaceful). Theta-band functional connectivity was quantified across four scalp regions (frontal, parieto-occipital, temporal, and central), with weighted phase lag index (wPLI) as the primary metric and phase-locking value (PLV) and coherence (Coh) as supplementary measures. Between-group comparisons (MCS vs. UWS) and within-group comparisons (each music condition vs. resting state) were performed to evaluate group differences in theta-band connectivity and its responsiveness to emotional music.

**Results:**

Between-group analyses showed that significant differences between MCS and UWS under rest and emotional music conditions were mainly located in frontal, parieto-occipital, and central regions (*p* < 0.05), whereas no consistent significant differences were observed in the temporal region. Overall, theta-band wPLI was higher in the UWS group than in the MCS group. Within-group analyses further demonstrated that, in the MCS group, frontal theta-band wPLI increased significantly after listening to sad (*p* = 0.019) and peaceful (*p* = 0.016) music, while no significant within-group changes were observed in the UWS group.

**Conclusion:**

In the context of emotional music stimulation, *θ*-band wPLI and its dynamic modulatory capacity may reflect differences in the reactivity of residual networks between MCS and UWS patients.

## 1 Introduction

Disorders of consciousness (DOC) patients are commonly subdivided into the minimally conscious state (MCS) and the unresponsive wakefulness syndrome (UWS). Compared with UWS, MCS patients generally exhibit a greater potential for neurological recovery. Current assessment of consciousness largely relies on standardized behavioral scales, among which the Coma Recovery Scale–Revised (CRS-R) is widely used as a core instrument (1). However, many patients have motor and/or language output impairments, subtle yet genuine signs of awareness may be overlooked, giving rise to so-called “covert consciousness” (2, 3). Therefore, relying solely on behavioral scales for clinical classification may lead to misdiagnosis, then disturb subsequent treatment planning (4). Accordingly, there is an urgent need for objective and reliable biomarkers that more directly reflect the brain’s intrinsic capacity for conscious processing. In recent years, neuroimaging and neurophysiological approaches—including positron emission tomography/computed tomography (PET/CT), magnetic resonance imaging (MRI), and electroencephalography (EEG) (5–7)—have shown encouraging progress. Among these, EEG offers high temporal resolution for capturing rapid brain dynamics and is portable and well suited for bedside application, making it an important tool for neurophysiological assessment.

Functional connectivity (FC) quantifies the statistical synchrony of neural activity between distinct brain regions. Common metrics include the phase-locking value (PLV), weighted phase lag index (wPLI), and coherence (Coh). These measures capture different aspects of synchrony. PLV primarily reflects the consistency of phase synchronization, but it is susceptible to volume conduction, which can produce spurious connections. Coh can be regarded as a frequency-domain correlation coefficient that characterizes the linear association between two signals at a given frequency, providing an intuitive index of band-specific coupling strength (8); however, it may likewise be confounded by volume conduction effect (9). In contrast, wPLI weights synchrony by the non-zero phase-lag component, thereby attenuating artificial correlations driven by near-zero phase differences. As such, wPLI can partially mitigate volume conduction–related bias and may more closely reflect genuine neural interactions (10). To reduce the influence of common noise sources and phase-covarying factors, we used wPLI as the primary FC metric, with PLV and Coh included as complementary measures for comparison.

One key prerequisite for consciousness is the capacity for rapid causal interactions among multiple specialized cortical regions (11). Accordingly, functional connectivity analyses are important for characterizing the electrophysiological features of DOC patients and may provide deeper evidence regarding mechanisms of large-scale functional integration in the human brain. To date, EEG-based FC studies in DOC patients have examined multiple frequency bands and brain regions, yet findings remain partially inconsistent. Prior work has suggested that, in DOC patients, alpha-band oscillations within the frontoparietal network are related to the level of consciousness, with similar associations also reported for gamma-band activity in parietal cortex (7). Other studies have reported that, compared with patients in the UWS, those in MCS show relatively better preservation of bilateral frontoparietal connectivity, and that recovery of consciousness may be accompanied by restoration of connectivity within this network (12). A TMS–EEG study found that theta-band reactivity was positively correlated with CRS-R scores (13), and additional evidence suggests that Coh of low-frequency cortical activity may serve as a relatively reliable marker of consciousness state (14). Moreover, direct analyses of local thalamic activity from central thalamic recordings in patients receiving deep brain stimulation (DBS) indicate that theta-related activity is associated both with functional recovery in DOC patients and with differences across levels of consciousness (15).

Music, as a common auditory stimulus, can modulate brain activity and cognitive processing and influence neural synchrony (16). Long-term musical training has also been shown to induce neuroplastic changes (17). During music listening, theta-band phase synchrony in right frontoparietal regions can increase (18), and musical training has likewise been reported to enhance inter-regional functional connectivity (19). Given that passive auditory stimulation is noninvasive, easy to implement, and does not depend on participants’ active engagement or comprehension, it may be more suitable than active paradigms for patients with severely impaired consciousness who are unable to follow task instructions. It also offers a potential approach for detecting and longitudinally tracking recovery of consciousness in brain-injured patients who cannot communicate effectively with the external environment.

Evidence further suggests that music can enhance theta–gamma phase–amplitude coupling in patients with DOC (11) and may strengthen connectivity between auditory networks and brain regions implicated in autobiographical memory (20, 21), indicating that music can elicit measurable electrophysiological changes. In parallel, multiple studies have shown that patients with DOC may retain partial sensitivity to emotionally salient stimuli (22–24), and that highly emotional sounds can evoke differential cortical responses in DOC (25). Therefore, emotionally valence musical stimulation may help reveal “covert consciousness” in this population.

Based on this evidence, we adopted an emotional music stimulation paradigm and focused on changes in theta-band EEG functional connectivity under stimulation, with the aim of examining how music influences brain network dynamics in DOC patients.

## 2 Objects and methods

### 2.1 Participants

This study included 48 DOC patients from the Department of Neurology and Department of Intensive Care Medicine of the First Affiliated Hospital of Anhui Medical University (Hefei, China), including 22 female patients and 26 male patients. Inclusion criteria include: (1) Patients diagnosed with UWS /MCS based on the CRS-R assessment, (2) aged between 18–80 years, (3) no sedative medication within 24 h before EEG recording, (4) no personal history of hearing impairment before onset (5) guardian signed informed consent. Exclusion criteria included: (1) known prior severe neurocognitive degenerative diseases (e.g., Alzheimer’s disease, Lewy body dementia), (2) skull defects, (3) combined with other psychiatric disorders, (4) significant EEG interference due to agitation or inability to cooperate, (6) Unstable condition, such as shock (systolic blood pressure <80 mmHg) or high fever (body temperature >38.5 °C). All of the patient’s treatment regimens continued as usual during the study period. The experimental procedure was approved by the Ethics Committee of the First Affiliated Hospital of Anhui Medical University (protocol code PJ2020-17-11) and was conducted in accordance with relevant guidelines and regulations. Each participant received a written informed consent.

### 2.2 Coma recovery scale assessment

The patient’s level of consciousness was evaluated before the experiment by two experienced physicians using the CRS-R, a tool recognized for its reliability in the behavioral assessment of DOC patients (26). The scale ranges from 0 to 23, with higher scores reflecting a greater level of patient awareness.

### 2.3 Musical stimuli

The study selected music in four different emotional categories: sad, fearful, happy and peaceful. The sad music was excerpted from the Chinese traditional folk piece *“Erquan Yingyue.”* The fearful music was selected from *“Ominous Presentiment,”* performed by Japanese composer Masami Ueda in *BIOHAZARD 3 LAST ESCAPE*. The happy music was taken from the Chinese traditional orchestral piece “Spring Festival Overture”. The peaceful music was selected from “One Day in Spring”, performed by the Swiss band Bandari. In the experiment, the researcher wore noise-canceling headphones in the patient’s ears and played the audio files through a computer with a uniform volume setting of 75 decibels. The experiment started with a 10-min resting baseline EEG recording during a no-music condition. Participants then listened to six-minute musical excerpts representing four emotional categories in randomized order. A two-minute rest interval was inserted between consecutive excerpts to minimize carry-over effects of neural activity.

### 2.4 EEG data acquisition and pre-processing

EEG recordings were obtained from 19 channels (O1, O2, P3, P4, Pz, T5, T6, C3, C4, Cz, T3, T4, F3, F4, Fz, F7, F8, Fp1, Fp2) using Ag/AgCl pin electrodes connected to a polygraph amplifier (EEG-1200C, Nihon Kohden, Japan) (27). The data were sampled at 200 Hz, with skin-electrode impedance maintained below 5 kΩ at all electrode sites. Preprocessing was performed using EEGLAB version 13.0b within the MATLAB 2022b environment (MathWorks Inc., Natick, MA, United States). A notch filter was applied to remove the 50 Hz powerline interference from the raw EEG signals. The signals were then band-pass filtered between 0.1 and 40 Hz (28). Independent component analysis (ICA) was utilized to identify and eliminate artifact-related components. ICA components were manually inspected and rejected by two experienced EEG researchers based on visual identification of characteristic artifact patterns, including muscle artifacts, ocular artifacts, and cardiac artifacts (29). The selected artifact-free epochs were re-referenced using an average reference, and any segments exceeding ±150 μV (30) were excluded from further analysis. Following preprocessing, no significant differences were observed in the retained data durations between the MCS and UWS groups across all conditions (all *p* > 0.05; see Supplementary Table S1 for details).

### 2.5 Within-region EEG functional connectivity analysis (Theta band: 4–8 Hz)

Electrodes were grouped into four anatomical scalp regions based on the international 10–20 system, and connectivity was computed only within each region: Frontal (Fp1, Fp2, F3, F4, F7, F8, Fz), Temporal (T3, T4, T5, T6), Central (C3, C4, Cz), and Parietal–Occipital (P3, P4, Pz, O1, O2). If a channel was not present in an individual dataset, it was excluded from the corresponding regional analysis, and connectivity matrices were computed using the available channels only.

#### 2.5.1 Theta-band spectral coherence (Coh)

Frequency-domain linear coupling within each region was quantified using magnitude-squared coherence (MSC) (31). For each within-region electrode pair (x,y) , MSC was estimated from Hann-tapered, epoch-wise FFTs by computing auto-spectra and cross-spectra and averaging them across epochs. Coh was defined as:


$$\mathrm{Co}h_{\mathrm{xy}}(f)=\frac{{|P_{\mathrm{xy}}(f)|}^{2}}{P_{\mathrm{xx}}(f)P_{\mathrm{yy}}(f)}$$

The Coh spectrum was then averaged over the theta band (4–8 Hz) to yield a single theta-band Coh value per electrode pair, forming a within-region Coh matrix for each participant.

#### 2.5.2 Theta-band phase synchronization: PLV and wPLI

To assess phase-based coupling, data were band-pass filtered in the theta range (4–8 Hz) using a FIR filter, and the analytic signal was obtained via the Hilbert transform. Instantaneous phase was defined as ∅(t)=arg(z(t)) , where z(t) denotes the analytic signal.

##### 2.5.2.1 Phase-locking value

For each electrode pair (x,y) , within each epoch the phase-difference time series was computed as $\Delta \emptyset_{\mathrm{xy}}(t)=\emptyset_{x}(t)-\emptyset_{y}(t)$ . PLV was defined as:


$$\mathrm{PL}V_{\mathrm{xy}}=|\frac{1}{T}\sum_{t=1}^{T}e^{\mathrm{i\Delta}\emptyset_{\mathrm{xy}}(t)}|$$

where is the number of samples per epoch (here *T* = 400). PLV was computed for each epoch and then averaged across epochs to yield a participant-level theta-band PLV for each electrode pair. PLV ranges from 0 to 1, with larger values indicating more consistent phase differences over time (32).

##### 2.5.2.2 Weighted phase lag index

To reduce the influence of zero-lag coupling potentially attributable to volume conduction or common reference effects, wPLI was additionally computed. Using the analytic signals $Z_{X}(t)\;$ and $Z_{y}(t)$ , the instantaneous cross-spectrum was:


$$S_{\mathrm{xy}}(t)=z_{X}(t)z_{y}^{*}(t)$$

Let Im(Sxy(t)) denote its imaginary component. wPLI was defined as:


ωPLIxy=∣E[Im(Sxy)]∣E[∣Im(Sxy)∣]

Where E[⋅] denotes averaging across time points (and subsequently across epochs). wPLI also ranges from 0 to 1 and emphasizes consistent non-zero phase-lag interactions. wPLI can be viewed as a weighted extension of the phase lag index (PLI) that reduces sample-size bias relative to PLI (9, 33).

### 2.6 Statistical analysis

Statistical analyses were conducted in MATLAB 2022b and SPSS 23.0 and comprised between-group and within-group comparisons. For between-group analyses, to investigate differences in FC between patients with MCS and UWS across music conditions, we performed network-based analyses using the Network-Based Statistic toolbox (NBS v1.2). Within a generalized linear model (GLM) framework, independent-samples *t* tests were applied to FC metrics within each brain region under different music conditions, with age and disease duration included as covariates in the design matrix to account for potential confounding effects. Family-wise error rate (FWER) was controlled at the network level (*p* < 0.05) using 5,000 nonparametric permutations to identify statistically significant connected subnetworks. For within-group analyses, to assess changes in FC before and after music intervention in the MCS and UWS groups, paired-samples *t* tests were performed for each patient comparing FC metrics between each of the four music conditions and the resting state. These analyses were also implemented in NBS v1.2; 5,000 nonparametric permutations were conducted with exchangeability blocks to accommodate the repeated-measures design, controlling FWER at the network level (*p* < 0.05) to detect significant subnetworks. In addition, for theta-band analyses, false discovery rate (FDR) correction was applied (*q* < 0.05) to control for multiple comparisons. Between-group comparisons of demographic and clinical baseline characteristics were performed in SPSS: chi-square tests were used for categorical variables, and Mann–Whitney U tests were used for continuous variables when normality assumptions were not met.

The primary hypotheses of this study were to examine between-group differences in theta-band wPLI between MCS and UWS groups under rest and emotional music conditions, focusing on the *a priori* selected regions of interest (ROIs): frontal and parietal lobes. These ROIs were chosen based on prior literature linking them to consciousness modulation (34, 35). The secondary hypotheses separately examined the changes in *θ*-band wPLI within the same ROIs in each group, after listening to music of different emotional valences, compared with the baseline condition.

## 3 Results

### 3.1 Demographic characteristics

A total of 48 DOC patients were included, comprising 21 cases of intracerebral hemorrhage and 27 cases of cerebral infarction; 26 were male and 22 were female. Patients were stratified into the MCS group (*n* = 32; 16 males and 16 females) and the UWS group (*n* = 16; 10 males and 6 females). The mean disease duration was (38.656 ± 9.927) days in the MCS group and (45.500 ± 17.752) days in the UWS group, with no significant between-group difference (z = −0.154, *p* = 0.878). The mean age was (63.031 ± 1.854) years in the MCS group and (64.500 ± 2.508) years in the UWS group, also showing no significant difference (z = −0.255, *p* = 0.799). In addition, no significant differences were observed between groups in gender (χ^2^ = 0.671, *p* = 0.413) or etiology (χ^2^ = 0.668, *p* = 0.414). Table 1 summarizes the demographic and clinical characteristics of all participants. See Supplementary Table S2 for details.

**Table 1:** Summarizes the demographic and clinical characteristics of all participants.

Variable	MCS group	UWS group	*p*-value
Number	32	16	
Age(year)	63.03 ± 1.85	64.50 ± 2.51	0.799
Gender			0.413
Male	16/32 (50%)	10/16 (62.5%)	
Female	16/32 (50%)	6/16 (37.5%)	
Etiology			0.414
CI	18/32 (56.25%)	9/16 (56.25%)	
CH	14/32 (43.75%)	7/16 (43.75%)	
Mean disease duration	38.66 ± 9.93	45.50 ± 17.75	0.878
CRS-R total score	11.75 ± 3.94	4.31 ± 1.62	<0.001

CI, cerebral infarction; CH, Cerebral hemorrhage; MCS, minimally conscious states; UWS, unresponsive wakefulness syndrome; CRS-R, Coma Recovery Scale-Revised.

### 3.2 Between-group differences

Under the resting condition, significant between-group differences were observed in theta-band wPLI in the frontal region (*p* = 0.019, FDR-corrected) and the parieto-occipital region (*p* = 0.046, FDR-corrected), with higher values in the UWS group than in the MCS group.

During sad music, theta-band wPLI in the parieto-occipital region (*p* = 0.016, FDR-corrected) and the central region (*p* = 0.042, FDR-corrected) was higher in the UWS group than in the MCS group. In addition, frontal theta-band wPLI was higher in the MCS group than in the UWS group (*p* < 0.001, FDR-corrected).

During fear music, theta-band wPLI in the central region (*p* = 0.011, FDR-corrected) and the parieto-occipital region (*p* = 0.015, FDR-corrected) was higher in the UWS group than in the MCS group. Meanwhile, frontal theta-band PLV was also higher in the UWS group than in the MCS group (*p* = 0.026, FDR-corrected).

During happy music, theta-band wPLI in the central region (*p* = 0.016, FDR-corrected) and the parieto-occipital region (*p* = 0.019, FDR-corrected) was higher in the UWS group than in the MCS group. Supplementary analyses further showed that frontal theta-band Coh and PLV were also higher in the UWS group than in the MCS group, suggesting consistent between-group differences in frontal theta oscillation–related connectivity across different connectivity metrics under happy music.

**Figure 1:**
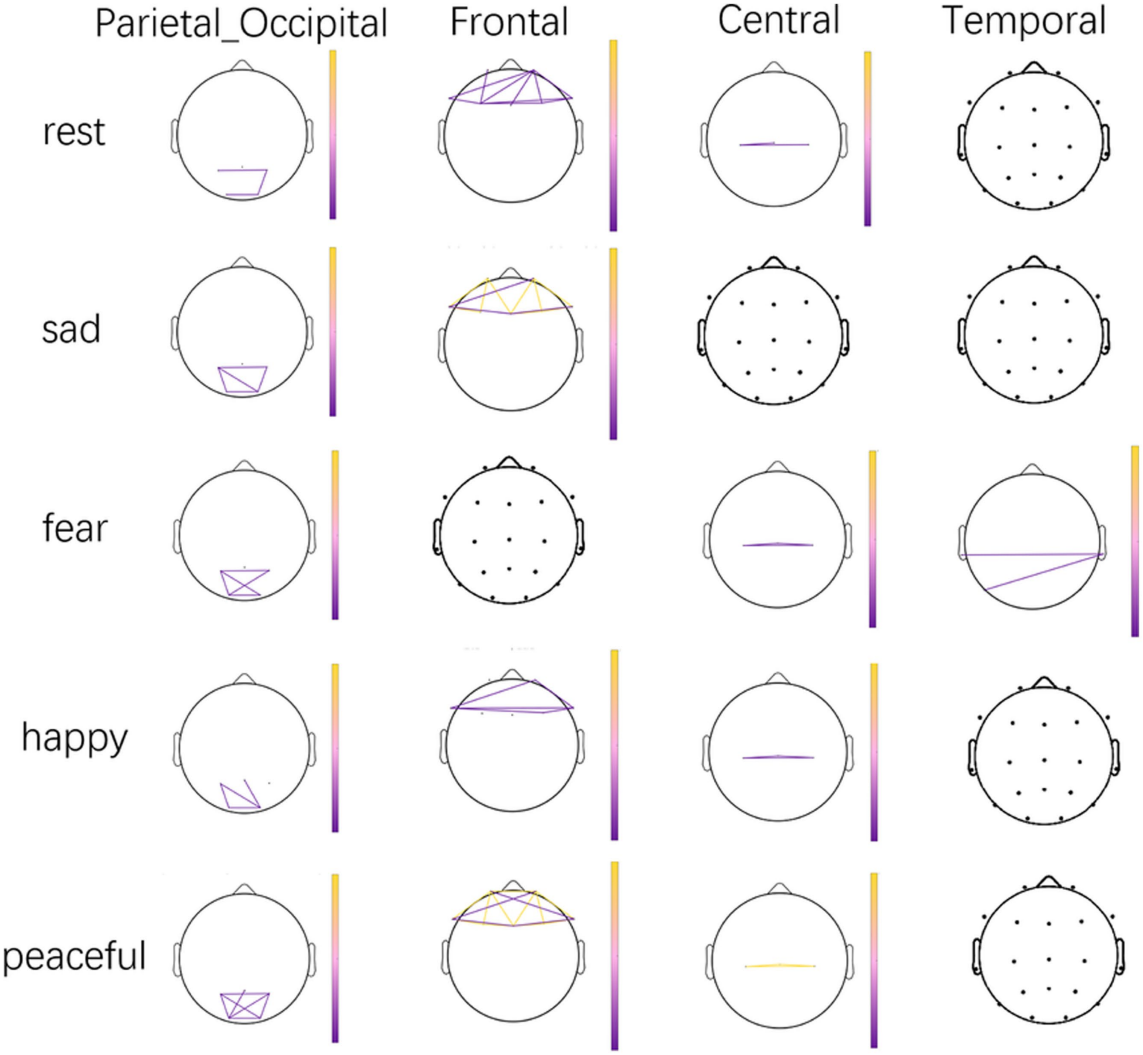
NBS-corrected significant theta-band weighted phase lag index (wPLI) edges differentiating the minimally consciousness state (MCS) and unresponsive wakefulness syndrome (UWS) groups across scalp regions. Yellow edges indicate higher wPLI in the MCS group than in the UWS group (MCS > UWS), whereas purple edges indicate higher wPLI in the UWS group than in the MCS group (UWS > MCS). Edges were identified using NBS permutation testing with network-level FWER control.

During peaceful music, the theta-band wPLI in the frontal and parieto-occipital regions exhibited distinct patterns of between-group differences, which originated from different functional connections within these regions. Specifically, some connections in the frontal and parieto-occipital regions showed significantly higher theta-band wPLI in the UWS group than in the MCS group (UWS > MCS; frontal: *p* = 0.003, FDR-corrected; parieto-occipital: *p* < 0.001, FDR-corrected); other connections in the frontal and central regions exhibited significantly higher theta-band wPLI in the MCS group than in the UWS group (MCS > UWS; frontal: *p* = 0.046, FDR-corrected; central: *p* = 0.006, FDR-corrected). This bidirectional pattern arises from distinct connection edges within the same regions. Supplementary analyses further indicated that frontal theta-band PLV and Coh were also higher in the UWS group than in the MCS group, supporting the robustness of frontal theta-band between-group differences under the peaceful music condition. The NBS-corrected theta-band wPLI edges showing significant differences between the MCS and UWS groups are shown in Figure 1, where yellow lines indicate MCS > UWS connections and purple lines indicate UWS > MCS connections for clear visualization of edge-specific directions. For reference, the detailed scalp electrode locations are provided in Supplementary Figure S1. The specific details can be found in Table 2.

**Table 2:** Between-group differences in theta-band weighted phase lag index (wPLI) between patients with MCS and UWS during resting state and emotion-specific music listening.

Music	Cortical region	Contrast	Number of significant edges	NBS-corrected P	FDR-corrected Q
Rest	Frontal	UWS-MCS	10	0.004	0.019*
	Parietal_Occipital	UWS-MCS	3	0.014	0.046*
	Central	UWS-MCS	3	0.018	0.052
Sad	Frontal	UWS-MCS	3	0.04	0.094
	Frontal	UWS-MCS	9	<0.001	<0.001*
	Parietal_Occipital	UWS-MCS	5	0.003	0.016*
	Central	MCS-UWS	2	0.012	0.042*
Fear	Central	UWS-MCS	3	0.001	0.011*
	Parietal_Occipital	UWS-MCS	5	0.003	0.015*
	Temporal	UWS-MCS	2	0.02	0.055
Happy	Frontal	UWS-MCS	5	0.033	0.084
	Parietal_Occipital	UWS-MCS	4	0.005	0.019*
	Central	UWS-MCS	3	0.002	0.016*
Peaceful	Frontal	MCS-UWS	4	0.015	0.046*
	Frontal	UWS-MCS	11	<0.001	0.003*
	Parietal_Occipital	UWS-MCS	7	<0.001	<0.001*
	Central	MCS-UWS	3	<0.001	0.006*

*Q < 0.05.

### 3.3 Within-group analysis

**Figure 2:**
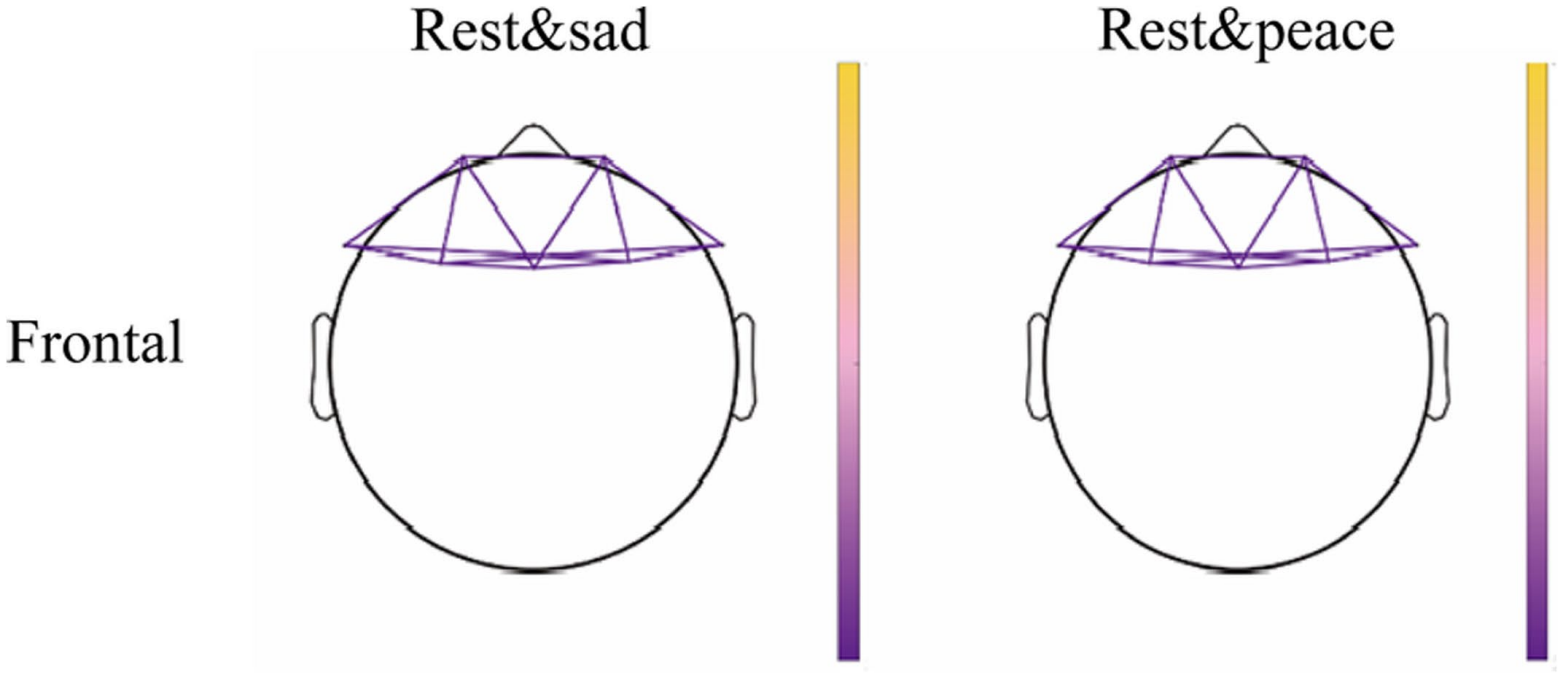
Significant theta-band wPLI edge changes in the MCS group during emotion-specific music relative to resting state across scalp regions (NBS-corrected). Yellow edges indicate higher wPLI in resting state than in music condition (Rest > Music), whereas purple edges indicate higher wPLI in music condition than in resting state (Music > Rest).

Within-group analyses showed that frontal theta-band wPLI in MCS patients increased significantly after listening to sad music (*p* = 0.019, FDR-corrected) and peaceful music (*p* = 0.016, FDR-corrected), whereas no significant changes were observed after the other music conditions. Figure 2 significant theta-band wPLI edge changes in the MCS group during emotion-specific music relative to resting state across scalp regions (NBS-corrected). The specific details can be found in Table 3.

**Table 3:** Theta-band wPLI changes in the MCS group during emotion-specific music relative to resting state.

Cortical region	Contrast	Number of significant edges	NBS-corrected P	FDR-corrected Q
Frontal	Sad-rest	13	<0.001	0.019*
Frontal	Peace-rest	13	<0.001	0.016*

*Q < 0.05.

## 4 Discussion

Using EEG data acquired during resting state and under emotion-specific music stimulation, this study compared between-group differences in theta-band functional connectivity between patients with MCS and UWS, as well as their connectivity response patterns to music. The between-group analyses revealed significant differences in theta oscillation–related connectivity, primarily involving frontal, parieto-occipital, and central regions. Overall, the UWS group exhibited higher wPLI. Notably, under sad and peaceful music, the frontal region (and the central region under peaceful music) showed between-group effects that deviated from the overall pattern, suggesting that emotional music may modulate local network synchrony associated with level of consciousness. Moreover, within-group analyses demonstrated that sad and peaceful music elicited a significant increase in frontal theta-band wPLI in the MCS group, whereas no comparable change was observed in the UWS group. Together, these findings advance the understanding of electrophysiological differences between MCS and UWS from the perspective of network synchrony and provide exploratory evidence relevant to assessing wPLI as a preliminary indicator for differentiating UWS from MCS.

MCS refers to a clinical condition in which patients exhibit minimal yet clearly discernible signs of consciousness and may demonstrate some degree of goal-directed behavior, whereas patients in UWS predominantly retain unconscious reflexive behaviors (36). Prior EEG studies have shown that UWS is characterized by more prominent delta/theta activity (37) and a stronger dominance of slow waves (38), whereas MCS tends to show relatively greater high-frequency components (39). In addition, DOC patients often exhibit relatively stronger connectivity in low-frequency bands (40). Accordingly, the present finding of overall higher theta-band wPLI in the UWS group compared with the MCS group is consistent with a slow-wave–dominated electrophysiological background.

Importantly, increased phase synchrony does not necessarily imply improved information integration. Disorders of consciousness are commonly accompanied by EEG slowing, thalamus–cortical dysconnectivity, reduced network differentiation, and decreased electrophysiological complexity (41). Diffuse brain injury may disrupt cortical and subcortical circuits, weaken inhibitory control, and promote disinhibition, resulting in a pathological state characterized by “increased synchrony but reduced complexity” (42). In this context, higher theta-band wPLI is more likely to reflect pathological hyper-synchronization or a low-complexity network pattern driven by shared slow rhythms (43), rather than enhanced effective information integration. Notably, under sad and peaceful music, the frontal region showed significant effects in both directions (UWS > MCS and MCS > UWS). This pattern suggests subnetwork-level reconfiguration within frontal circuits rather than a unidirectional change, and further implies that frontal networks may retain some capacity to differentiate emotional music features and respond to them. Moreover, we performed supplementary analyses using PLV and Coh; in selected conditions and regions, these metrics showed between-group differences in the same direction as wPLI, providing additional support for the robustness of the primary findings.

Spatially, the between-group differences were mainly concentrated in frontal, parieto-occipital, and central regions, whereas no consistent significant differences were observed in the temporal region. In general, frontal, parieto-occipital, and central areas are more frequently linked to higher-order cognitive control, multisensory integration, and sensorimotor-related processing, which typically rely on broad network-level coordination. By contrast, the temporal region is primarily involved in auditory processing (44). Given that UWS is characterized largely by preserved low-level reflexive activity, whereas MCS patients can still exhibit some degree of goal-directed responses to external stimuli and retain aspects of higher cognitive function, regions supporting higher-order integration may be more sensitive to differences in consciousness-related processing capacity. Meanwhile, both UWS and MCS patients may retain basic auditory pathways and primary auditory responses to some extent, which could render temporal-region between-group differences less pronounced than those observed in integrative fronto–parieto-occipital and central regions. It should be noted that the 19-channel scalp EEG used in the present study has inherent limitations in spatial coverage and spatial resolution, which may reduce sensitivity for detecting temporal-lobe differences. Future work could incorporate source-level analyses and multimodal approaches such as MEG or fMRI to more rigorously evaluate the relative contributions of temporal auditory networks and higher-order integrative networks across different levels of consciousness.

In this study, we compared condition-specific connectivity changes in patients with MCS and UWS during music with different emotional valences. We found that, in the MCS group, frontal theta-band wPLI changed significantly after listening to sad and peaceful music, whereas no comparable within-group effects were observed in other regions or under happy and fear music. This within-group change aligns with the between-group results observed in the prefrontal region: the more pronounced increase in wPLI in patients with MCS under sad and calm music may, to some extent, have contributed to the reversal in the direction of between-group differences under these conditions. Taken together, these findings suggest that music stimuli elicit group-specific network responses in the prefrontal cortex and indicate that MCS patients may retain a certain capacity for dynamic modulation in response to emotion-related stimuli. Prior work indicates that frontal regions are involved in emotional experience and regulation (45), and that the prefrontal cortex plays a key role in pleasure elicited by music listening (46). Theta oscillations have also been implicated in processing emotional stimuli (47), with particularly prominent theta activity linked to the anterior cingulate cortex (48), potentially providing a neurophysiological basis for integrating multimodal information with contextual/episodic memory. In healthy participants, music listening has been associated with increased prefrontal theta power (49), and such emotional modulation appears to depend not only on valence but also on arousal (50). Our findings are broadly consistent with evidence that music can modulate prefrontal theta-related activity; notably, pleasurable frontal responses may also occur in the context of sad music (46).

Evidence from DOC cohorts further suggests that music listening can enhance connectivity across multiple regions and induce measurable connectivity changes (51). In addition, emotional processing may be partially preserved in both MCS and UWS (39), and stimuli related to preferred music have been reported to increase theta-band EEG amplitude in DOC patients (52). Taken together, we speculate that MCS patients may retain a relatively sensitive capacity for frontal network modulation in response to emotional cues, potentially reflecting residual auditory processing and the recruitment of frontal theta mechanisms related to sustained attention and/or emotional experience. Moreover, compared with fear and happy music, sad and peaceful music typically elicit lower arousal, which may facilitate more stable attentional engagement and thereby promote more consistent phase-lagged coupling in the frontal theta band—an effect that can be captured by wPLI. This is consistent with findings in healthy individuals, where slow-tempo, low-arousal music induces increased frontal theta power, associated with sustained attention and emotional integration; whereas fast-tempo, high-arousal music may elicit stronger physiological activation, leading to elevated power in high-frequency bands (beta/gamma) and desynchronization or reallocation of functional connectivity in certain frequencies or regions (53). In line with this interpretation, a previous study reported increased fronto–parietal–occipital PLV in DOC patients who responded to transcranial direct current stimulation (tDCS) compared with non-responders (54). The present frontal theta connectivity changes observed after low-arousal emotional music (sad/ peaceful) show some phenomenological similarity, suggesting that music-based stimulation may have potential value in arousal-modulation research in DOC.

By contrast, no significant within-group changes were observed in the UWS group across music conditions. This pattern may indicate that the higher theta-band connectivity observed in UWS in between-group analyses is more consistent with pathological hyper-synchronization in a slow-wave–dominated background, rather than flexible modulation by external emotional auditory cues. Overall, these findings suggest that passive emotional auditory stimulation may provide complementary information for probing residual processing capacity in DOC assessment and intervention studies.

## 5 Conclusion

This study compared theta-band EEG functional connectivity in MCS and UWS patients during resting state and under emotion-specific music stimulation. Detectable between-group differences in functional connectivity were observed, primarily in frontal, parieto-occipital, and central regions; during resting state and under selected music conditions, wPLI was higher in the UWS group than in the MCS group. In addition, within-group analyses indicated that frontal theta-band wPLI changed significantly in the MCS group after listening to sad and peaceful music, whereas no significant within-group changes were observed in the UWS group. Collectively, these findings suggest that theta-band functional connectivity and its modulability during emotional music stimulation may reflect differences in residual network responsiveness in patients with disorders of consciousness, offering preliminary evidence that warrants further exploration of emotion-based music paradigms in consciousness state assessment. Future studies could validate its ability for clinical classification of consciousness state through machine learning approaches.

## 6 Limitation

There are several limitations in this paper. First, sample size may limit power and stability; larger, better-balanced cohorts and matching sensitivity analyses are needed. Secondly, Scalp-EEG connectivity without source reconstruction limits spatial inference; future work should add MRI-informed source localization and large-scale network interpretation. Thirdly, Findings reflect short-term physiological changes and do not establish clinical efficacy; longitudinal repeated-measures studies with outcomes (e.g., CRS-R) are required. Fourth, this study did not conduct quantitative diagnostic efficacy evaluation (such as ROC/AUC analysis or machine learning classifiers), so it is unable to fully verify the clinical utility of *θ*-band wPLI as a potential indicator for distinguishing MCS from UWS. Future studies should adopt appropriate validation methods to supplement this limitation. Larger samples could enhance stability in future work.

## Data Availability

The datasets presented in this article are not readily available because the data from this study are not publicly available due to privacy restrictions related to study participants. Researchers with legitimate requests may contact the corresponding author (email: [nicuay@163.com]) to obtain access. Requests to access the datasets should be directed to nicuay@163.com.
