# Supplementary material for: Theta-band EEG functional connectivity during emotional music in disorders of consciousness: wPLI differences between MCS and UWS
Source: Front Neurol. 2026 Mar 19;17:1777525. doi: 10.3389/fneur.2026.1777525 (PMC13043408; doi:10.3389/fneur.2026.1777525)

Supplementary Table S1. Retained EEG data duration (mean ± SD (minutes)) after preprocessing for each condition in the minimally conscious states (MCS) and unresponsive wakefulness syndrome (UWS) groups, with between-group comparisons

condition	MCS (min)	UWS (min)	P-value
Rest	8.69 ± 2.21	9.88 ± 2.82	0.167
Sad	4.91 ± 1.15	5.38 ± 1.46	0.178
Fear	4.50 ± 1.11	5.05 ± 1.11	0.057
Happy	4.68 ± 1.32	4.84 ± 0.89	0.498
Peace	4.58 ± 1.48	4.96 ± 1.40	0.330

Supplementary Table S2 Demographic information and clinical data of the DOC patients

ID	Gender	Age	Etiology	CRS-R	A	Vi	M	Ve	C	Ar	Diagnosis
1	Male	40	CH	14	2	3	5	1	1	2	MCS
2	Male	68	CI	10	1	1	5	1	0	2	MCS
3	Male	51	CI	2	0	0	0	0	0	2	UWS
4	Male	57	CH	4	0	0	3	1	0	0	MCS
5	Male	53	CH	4	0	1	0	1	0	2	UWS
6	Male	73	CI	3	0	0	2	0	0	1	UWS
7	Male	71	CI	4	0	0	2	1	0	1	UWS
8	Male	48	CH	11	2	1	5	1	0	2	MCS
9	Female	74	CH	6	1	1	2	1	0	1	UWS
10	Female	58	CI	8	0	2	2	1	1	2	MCS
11	Female	72	CH	7	1	0	5	1	0	0	MCS
12	Male	71	CI	5	1	0	1	1	0	2	UWS
13	Female	58	CI	2	0	0	1	1	0	0	UWS
14	Female	55	CI	14	2	3	5	1	0	3	MCS
15	Male	68	CI	19	3	3	5	3	2	3	MCS
16	Male	51	CI	2	0	0	0	0	0	2	UWS
17	Male	57	CH	4	0	0	3	1	0	0	MCS
18	Male	53	CH	12	2	3	2	1	1	3	MCS
19	Male	73	CI	14	2	3	5	1	0	3	MCS
20	Female	74	CH	6	1	1	2	1	0	1	UWS
21	Female	72	CH	8	1	0	5	1	0	1	MCS
22	Male	71	CI	5	1	0	1	1	0	2	UWS
23	Female	58	CI	13	4	3	1	1	1	3	MCS
24	Female	55	CI	14	2	3	5	1	0	3	MCS
25	Female	74	CI	10	1	1	5	1	0	2	MCS
26	Female	74	CH	3	0	0	2	1	0	0	UWS
27	Female	74	CI	9	2	3	0	1	0	3	MCS
28	Female	58	CI	10	1	1	5	1	0	2	MCS

ID	Gender	Age	Etiology	CRS-R	A	Vi	M	Ve	C	Ar	Diagnosis
29	Female	74	CI	14	2	4	5	1	0	2	MCS
30	Male	71	CI	13	3	2	2	1	2	3	MCS
31	Female	74	CI	3	0	0	2	1	0	0	UWS
32	Male	74	CH	14	2	3	5	1	0	3	MCS
33	Male	74	CH	16	3	3	5	1	1	3	MCS
34	Female	74	CI	19	3	4	5	2	2	3	MCS
35	Male	74	CH	16	3	3	5	1	1	3	MCS
36	Male	74	CH	20	4	4	5	2	2	3	MCS
37	Male	48	CH	15	2	3	5	1	1	3	MCS
38	Male	53	CH	6	0	1	2	1	0	2	UWS
39	Male	48	CH	11	2	1	5	1	0	2	MCS
40	Female	74	CI	9	2	3	0	1	0	3	MCS
41	Male	53	CI	6	0	1	2	1	0	2	UWS
42	Female	74	CH	6	1	1	2	1	0	1	UWS
43	Female	58	CI	10	1	1	5	1	0	2	MCS
44	Female	57	CI	11	2	3	2	2	0	2	MCS
45	Male	64	CH	8	2	2	2	0	0	2	MCS
46	Female	46	CI	11	2	3	2	1	1	2	MCS
47	Female	67	CI	8	0	2	2	1	1	2	MCS
48	Male	57	CH	6	1	1	2	0	0	2	UWS

CI, cerebral infarction; CH, Cerebral hemorrhage; CRS-R, Coma Recovery Scale-Revised; CRS-R subscales: A, auditory function; Vi, visual function; M, motor function; Ve, verbal; C, communication; Ar, arousal; MCS, minimally conscious states; UWS, unresponsive wakefulness syndrome

Figure S1 Scalp electrode positions.

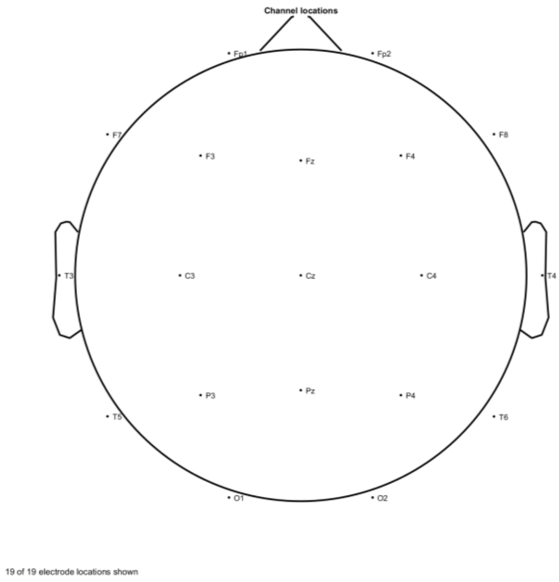

Supplement: Supplementary file 1 [file Data_Sheet_1.PDF]
